# Awareness of disease extent and perceived palliative treatment goals among oncology patients: a multicenter study

**DOI:** 10.1038/s41598-026-55911-0

**Published:** 2026-06-04

**Authors:** Manar Hamed, Hend M. Hamdy Rashed Elkalla, Waled Bahaj, Rehab Hassan, Aalaa M. Abdou, Samar Horya, Basma Rezk Farouk, Rabab M. Farghaly, Eman Mahmoud Shoaib, Sanaa Saber Abd El-raheem, Sally Abdelfattah Elkhamisy, Mayada Fawzy Sedik, Ghada Mohamed Ahmad Zahir, Sara Mahmoud El Zayat, Hadeel Gamal Elghamery, Mohamed Roshdy Mohamed Al Jamal, Omar Hamdy, Mohamed M. Hegazi

**Affiliations:** 1 Medical Oncology Unit, Internal Medicine Department, Faculty of Medicine, Oncology Centre, Mansoura University, Mansoura, Egypt; 2 Clinical Oncology and Nuclear Medicine Department, Faculty of Medicine, Mansoura University, Mansoura, Egypt; 3 Division of Hematology Oncology, University of Louisville, Brown Cancer Center, Louisville, USA; 4 Medical Oncology and Hematology Department, South Egypt Cancer Institute, Assiut University, Asyut, Egypt; 5 El Zaitoun Specialized Hospital, Ministry of Health and Population, Cairo, Egypt; 6 Clinical Pharmacist, Oncology Center, Mansoura University, Mansoura, Egypt; 7 Biostatistics and Cancer Epidemiology Department, South Egypt Cancer Institute, Assiut University, Asyut, Egypt; 8 Clinical Oncology Department, Mansoura New General Hospital, Mansoura, Egypt; 9 Surgical Oncology Department, Oncology Center, Mansoura University, Mansoura, Egypt

**Keywords:** Palliative care, Quality of life, Treatment goals, Cancer, Diseases, Health care, Medical research, Oncology

## Abstract

In Egypt, approximately 134,000 new cancer cases and over 30,000 deaths are recorded annually. Unfortunately, a considerable proportion of patients present with an advanced stage. This patient population often faces a misconception that if they understand their disease stage and treatment goals, it will increase their anxiety. This study assessed Egyptian patients’ perceptions of their treatment objectives and the factors influencing their understanding. A survey-based study was conducted involving 487 Stage IV solid malignancy patients receiving palliative care at four Egyptian cancer centers. The survey assessed the patients’ awareness of their cancer diagnosis, stage, and treatment goals. Demographic data were collected. Responses were compared with medical records, and a total knowledge score (TKS) was calculated. A cutoff of ≥ 75% was set for satisfactory knowledge. Of the 487 patients, 46 (9.4%) denied having cancer and were excluded. Among the remaining 441 patients, despite 85.5% accurately knowing their tumor site, only 20.4% defined their exact tumor stage, and only 12% understood that the treatment was palliative rather than curative, while 28.9% thought that their tumors were benign. Collectively, 12% demonstrated a satisfactory knowledge level. The most significant factor that correlated positively with TKS was the education level (r_s_ = 0.311, *P* < 0.001). Illiterate/primary-level education patients were 2.28 times more likely to have misconceptions. A substantial percentage of cancer patients demonstrated insufficient knowledge about their disease nature and treatment goals, especially those with lower education levels. These findings highlight the importance of improving patient education and physician-patient communication to help improve the process of shared decision-making.

## Introduction

 Each year in Egypt, there are approximately 134,000 cancer cases diagnosed, leading to more than 30,000 deaths annually^1^. Regrettably, a considerable number of cases are diagnosed in advanced stages of cancer, with a prevalence of Stage IV lung, colon, and breast cancers of 58%, 28.6%, and 10.2%, respectively^2–4^.

Metastatic cancer frequently has a significant symptom burden, an inferior quality of life (QOL), and an unfavorable prognosis. Various treatment modalities are offered to patients with metastatic disease^5–8^. The primary objective of treatment at this stage is palliative to alleviate symptoms, temporarily control the disease, enhance QOL, and prolong survival. Unfortunately, not all patients grasp the purpose of their treatment goals^5^.

While commencing cancer therapy, patients’ perception of their treatment goal is a core element of the informed consent process^5^. Nonetheless, numerous studies have revealed that individuals with advanced cancer commonly have significant misunderstandings about their disease stage and treatment objectives^9–18^.

Culturally, it is still widely believed in Egypt that withholding the cancer diagnosis from the patient reduces psychological distress. Conversely, a previous study conducted in two Egyptian cancer centers confirmed that patients who are unaware of their cancer diagnosis do not experience a lower level of anxiety and depression, nor do they have a better quality of life^19^.

We conducted this study to assess Egyptian patients’ perceptions of their treatment goals and the potential factors that influence these perceptions.

## Methods

The study is a cross-sectional, questionnaire-based study that included patients diagnosed with Stage IV solid malignancies who were receiving palliative cancer treatment and were older than 18 years. Patients were recruited from the oncology outpatient clinics of the Mansoura University oncology center, the Mansoura University Clinical Oncology Department, the Assiut University South Egypt Cancer Institute, and El Zaitoun Specialized Hospital. Patients with hematologic malignancies, cognitive impairment, or sensory deficits were excluded.

Data were collected between January 2021 and December 2021. The study protocol was approved by the institutional research board (IRB) of the faculty of Medicine, Mansoura University (R.21.06.1366.R1), and the Research Ethics Committee (REC) of the Egyptian Ministry of Health and population (7-2022/06). It was carried out in compliance with the Declaration of Helsinki. Informed written consent was obtained from the patients. The questionnaire was self-reported by patients who can read. Those who could not read were interviewed by the physician. The primary outcome was to assess the patients’ perception and understanding of the treatment goal. Adequate understanding was defined as patients’ recognition that treatment intent was non-curative (palliative), regardless of their expectations regarding potential survival prolongation.

Estimating the percentage of patients with sufficient knowledge about their condition and treatment objectives was used to determine the sample size. The minimum necessary sample size was determined to be 384 patients, assuming an estimated prevalence of sufficient knowledge of 50% (to yield the maximum sample size in the absence of prior precise estimations), a confidence level of 95%, and a margin of error of 5%. A target sample of at least 460 participants was obtained by increasing the sample size by about 20% to account for potential non-response, incomplete questionnaires, or exclusion of patients who denied their diagnosis.

The information sought from patients included details such as the primary site of malignancy, stage, treatment duration, perception regarding the curative nature of treatment, and beliefs about the efficacy in extending life or alleviating symptoms related to cancer. Additionally, patients provided basic demographic information such as age, gender, marital status, area of residence, and education level. We assessed patients’ awareness regarding cancer type, stage, and duration of treatment and compared their answers with medical records. If the patient answered the 1st question (“ Do you suffer from a tumor? “) with no, the patient was given a score of 0 and did not need to complete the questionnaire. For each correctly answered question, the patient was given a score of 1, except in question Q6, as multiple answers were allowed. The total score was 14. The Total Knowledge Score (TKS) was analyzed both as a continuous variable and as a categorical variable using a predefined cutoff to facilitate clinical interpretation. A satisfactory level of knowledge, a cut-off of 75%, was set^20,21^.

### Statistical analysis

Data were analyzed using IBM Statistical Package for the Social Sciences (SPSS) software, IBM Corp., Released 2019. IBM SPSS Statistics for Windows, Version 26.0. Armonk, NY: IBM Corp, https://www.ibm.com/products/spss-statistics. Qualitative data were expressed as absolute frequency (N) and relative frequency (%). Phi (ϕ) was used to assess the strength of association. Quantitative data were initially tested for normality using Shapiro-Wilk’s test, with data being normally distributed if *P* > 0.050. The presence of significant outliers (extreme values) was tested by inspecting boxplots. Data were expressed as mean ± SD if normally distributed or median (Q1-Q3) if not. The chi-square test and Fisher’s exact test were used to assess the association between two nominal variables. The chi-square test was used if the expected count in all cells ≥ 5; otherwise, Fisher’s exact test was used. The Mann-Whitney U-test was used to compare non-normally distributed quantitative data between two groups. Spearman’s correlation was used to assess the direction and strength of association between two quantitative variables. Binary logistic regression was used to identify factors associated with an unsatisfactory knowledge level. Variables were selected based on clinical relevance and univariate analysis results. Adjusted odds ratios (AORs) with 95% confidence intervals (CIs) were reported. For any of the used tests, results were considered statistically significant if the *P*-value ≤ 0.050. Relevant graphical representations were used whenever needed.

## Results

This study included 487 patients with Stage IV cancer undergoing palliative treatments. Among them, 46 (9.4%) denied having a cancer diagnosis and were excluded from the survey. The final analysis included 441 patients.

**Fig. 1:**
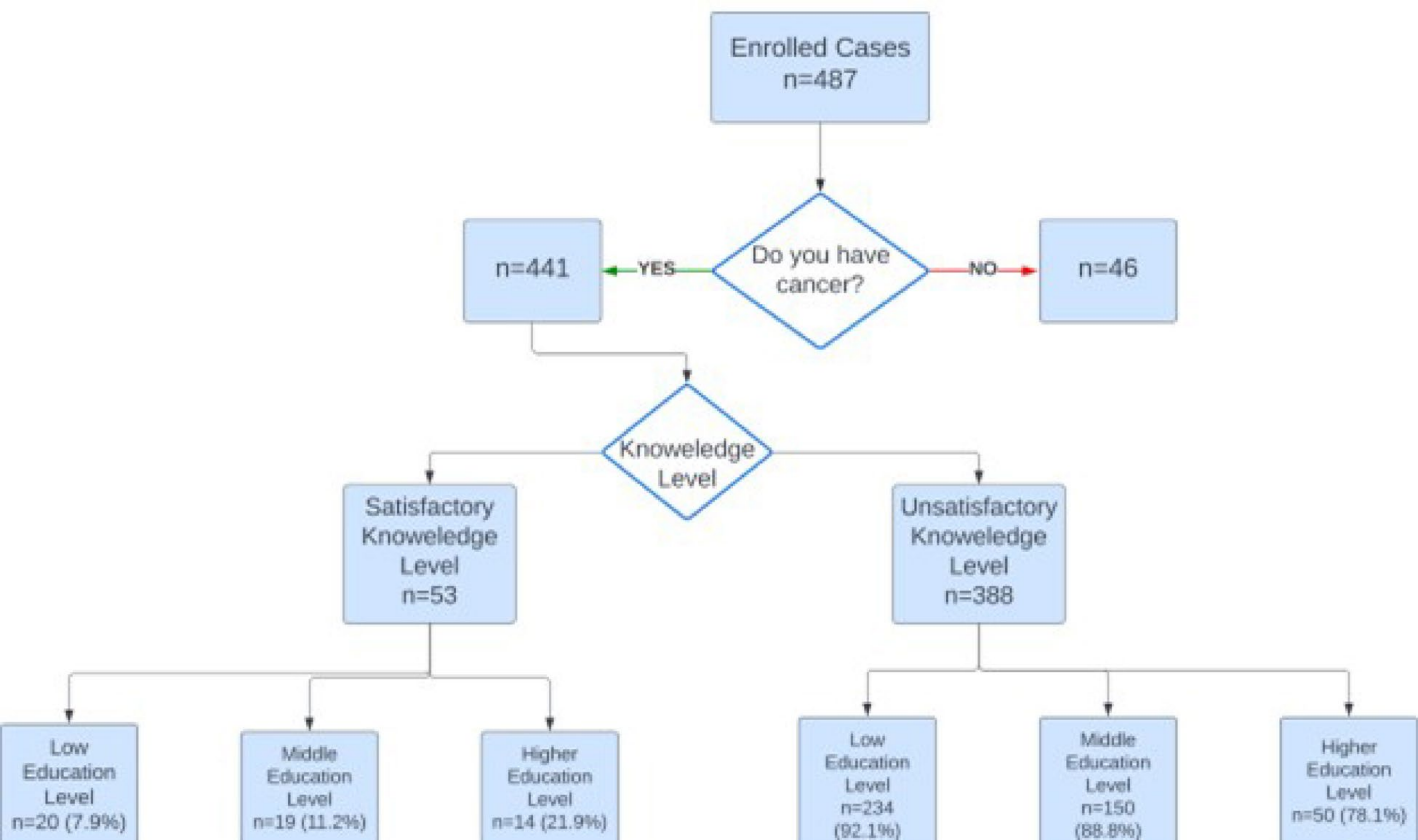
A flow algorithm showing the total number of patients in the study, addressing the knowledge level.

Regarding baseline comprehension, only 53 patients (12%) demonstrated a satisfactory level of knowledge (≥ 75%). When exploring the education level, only 21.9% of those with a higher education level (college and postgraduate students) showed a satisfactory level of knowledge, compared with 11.2% of middle school graduates, and 7.9% of the lower education level group (Fig. 1; Table 1).

**Table 1:** Baseline clinical and demographic characteristics.

Characteristic	*N* (%)
Sex	
Male	159 (32.6%)
Female	328 (67.4%)
Age (years)	
< 40	98 (20.1%)
40–60	234 (48%)
> 60	155 (31.8%)
Marital status	
Single	26 (5.3%)
Married	387 (79.5%)
Divorced	13 (2.7%)
Widow	61 (12.5%)
Educational level	
Illiterate	214 (43.9%)
Primary school	40 (8.2%)
Middle	100 (20.5%)
Technical	69 (14.2%)
University graduate	61 (12.5%)
Postgraduate	3 (0.6%)
Residence	
Rural	334 (68.6%)
Urban	153 (31.4%)
Cancer type	
Breast	188 (38.6%)
Gynecologic	41 (8.4%)
Urinary	56 (11.5%)
Gastro-intestinal	111 (22.8%)
Lung	53 (10.9%)
Others	38 (7.8%)
Actual current treatment	
Chemotherapy	352 (72.3%)
Radiotherapy	24 (4.9%)
Hormonal	89 (18.3%)
Target therapy	24 (4.9%)
Actual treatment duration	
< 3 months	46 (9.4%)
3–6 months	231 (47.4%)
> 6–12 months	118 (24.2%)
> 12 months	92 (18.9%)

In addition, when answering survey questions, 28.9% of the patients believed that the tumor they had was not malignant. A significant portion of patients, 85.5%, correctly reported the tumor site. Surprisingly, only 20.4% were aware that the disease stage was Stage IV. While 94% acknowledged receiving treatment, only 12% of the patients understood that the aim of treatment was not for a cure. When addressing treatment aims, 68.4% of patients believed that their treatment goals were to alleviate symptoms and to improve quality of life. The median total knowledge score (TKS) was 9 (64.3%), with only 10.9% having a satisfactory level of knowledge (Table 2).

**Table 2:** Knowledge questionnaire results.

Question	Patients’ answers	Correct answers *N* (%)
Q1 (Do you suffer from a tumor? ), yes	441 (90.6%)	441(90.6%)
Q2 (What is the tumor type you have? )		313 (70.9%)
Benign	37 (8.3%)	
Malignant	313 (70.9%)	
Don’t know	91 (20.6%)	
Q3 (Where is the site of the tumor you have? )		377 (85.5%)
Breast cancer	172 (39%)	
Gynecological	36 (8.1%)	
Urinary tract	43 (9.7%)	
Gastrointestinal tract	97 (21.9%)	
Lung	40 (9%)	
Other	53 (12%)	
Q4 (Do you know your tumor stage? )		90 (20.4%)
Stage 1	22 (4.9%)	
Stage 2	22 (4.9%)	
Stage 3	13 (2.9%)	
Stage 4	90 (20.4%)	
Don’t know	293 (66.4%)	
Q5 (Are you currently under treatment for your tumor? )		415 (94.1%)
Yes	415 (94.1%)	
No	26 (5.8%)	
Q6 (What is the type of treatment that you are receiving? )		
Chemotherapy	269 (60.9%)	354 (80.3%)
Radiotherapy	22 (4.9%)	370 (83.9%)
Hormonal therapy	55 (12.4%)	360 (81.6%)
Target therapy	23 (5.2%)	359 (81.4%)
Don’t know	65 (14.7%)	
Q7 (How long have you been under this line of treatment? )		222 (50.3%)
< 3 months	59 (13.3%)	
3–6 months	68 (15.4%)	
6–12 months	78 (17.6%)	
> 12 months	200 (45.3%)	
Don’t know	36 (8.1%)	
Q8 (Did you receive brochures about your disease? )		128 (29%)
Yes	128 (29%)	
No	313 (70.9%)	
Q9 (Does your treatment have curative intent? )		55 (12.4%)
Yes	172 (39%)	
No	55 (12.4%)	
Don’t know	214 (48.5%)	
Q10 (Do you think your treatment will prolong your life? )		43 (9.7%)
Yes	142 (32.1%)	
No	43 (9.7%)	
Don’t know	256 (58%)	
Q11 (Do you believe your treatment will palliate your symptoms and improve your quality of life? )		302 (68.4%)
Yes	302 (68.4%)	
No	48 (10.8%)	
Don’t know	91 (20.6%)	
Total knowledge score median [minimum-maximum] (Q1-Q3)	9 [0–12] (6–10)	
Total knowledge % median [minimum-maximum] (Q1-Q3)	64.3 [0-85.7] (42.9–71.4)	
Satisfactory level (> 75%)	53 (10.9%)	

**Fig. 2:**
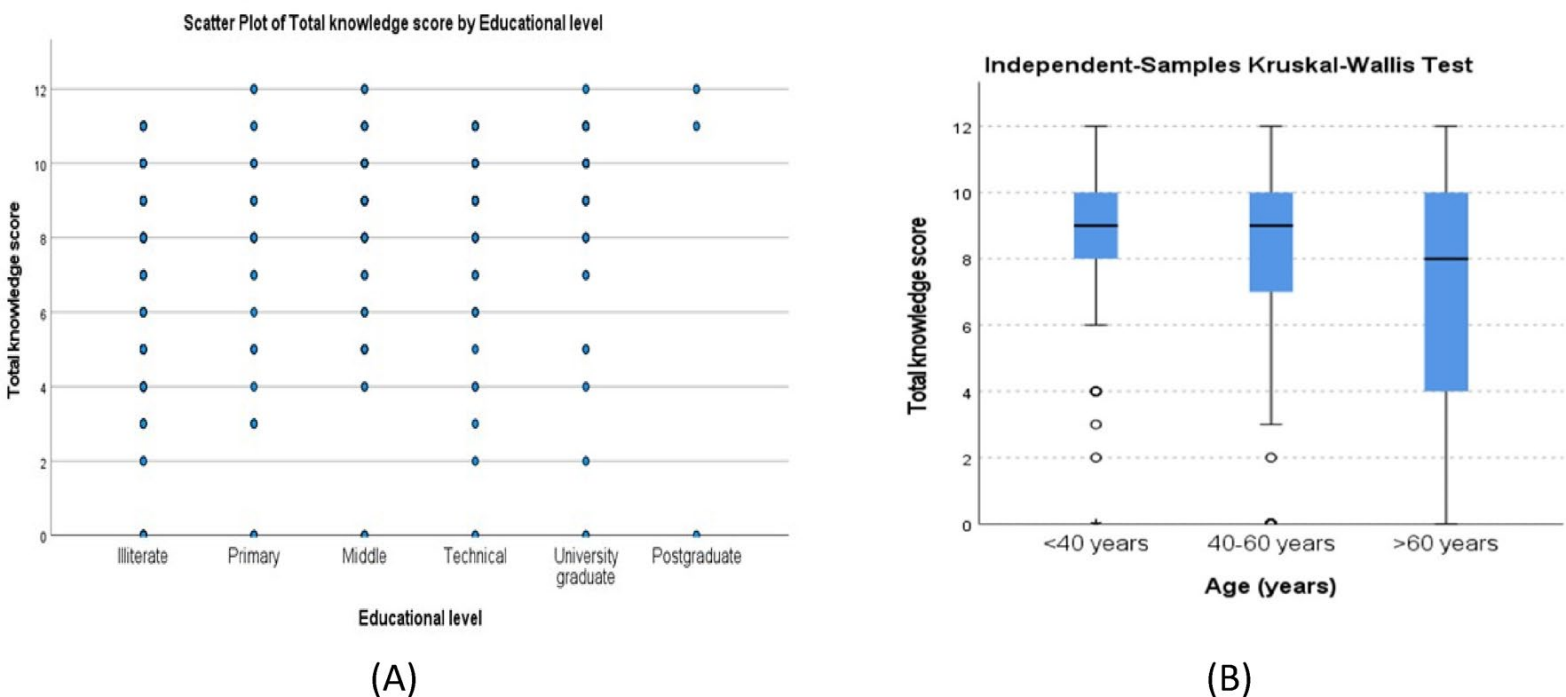
(**A**) A scatter plot of total knowledge score by educational level of illiterate, primary, middle, technical, university graduate, and postgraduate levels. Blue dots represent the correlation between educational level and the total knowledge score. (**B**) A box plot of the total knowledge score by patients’ age < 40, 40–60, and > 60 using the independent samples Kruskal-Wallis test.

A statistically significant positive correlation of medium strength was observed between TKS and education level (Spearman’s correlation coefficient = r_s_ = 0.311, *P* < 0.001) (Fig. 2a). Similarly, an independent samples Kruskal-Wallis test shows TKS across age groups (Table 3; Fig. 2b).

**Table 3:** Correlation between total knowledge score and patients’ characteristics.

Characteristic	*r*_s_	*p*-value
Education level	0.311	**< 0.001**
Age	-0.195	**< 0.001**

**Fig. 3:**
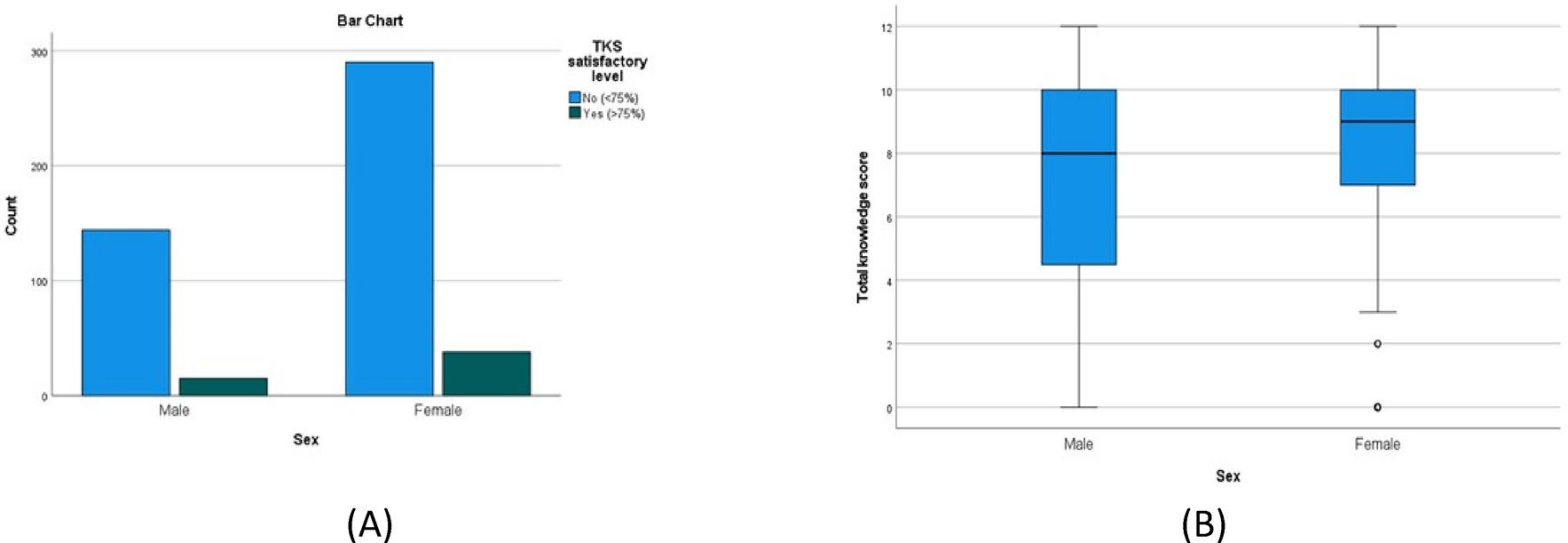
(**A**,**B**) A bar chart and a box plot that show the proportion of patients with and without a satisfactory level of knowledge in males and females.

**Fig. 4:**
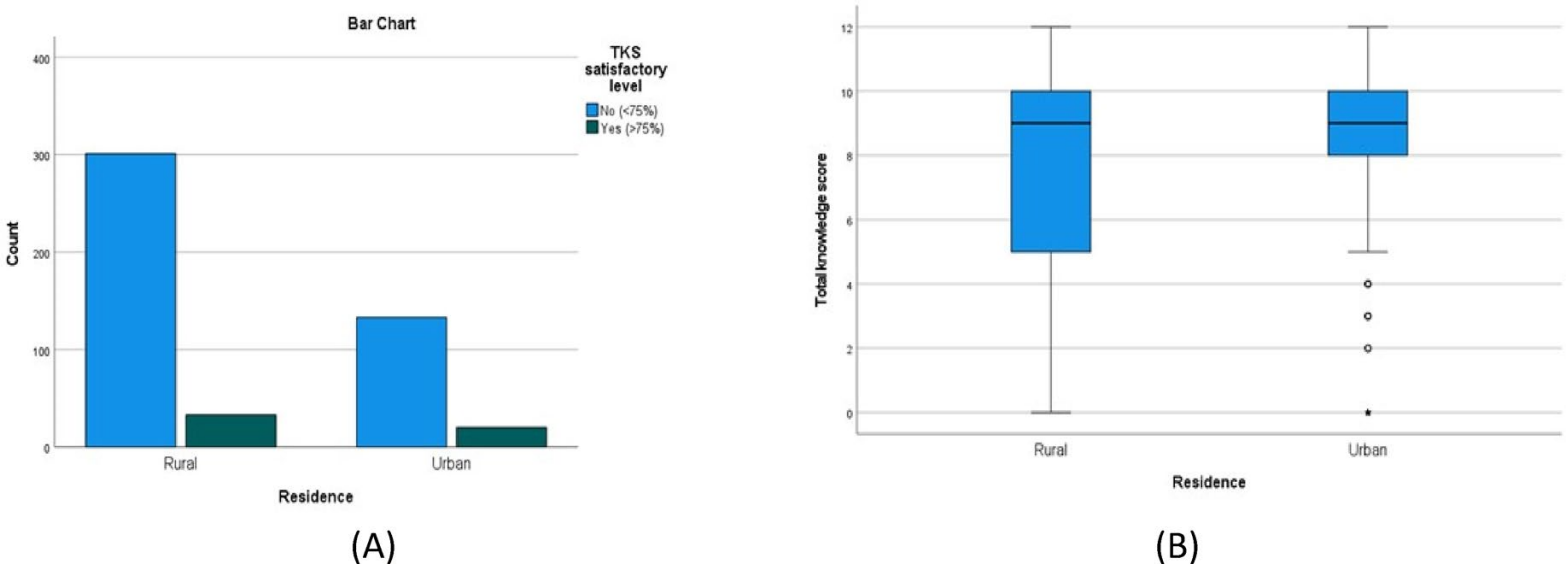
(**A**,**B**) A bar chart and a box plot that show the total knowledge score by residence. The green bar is satisfactory, and the blue bar is unsatisfactory.

**Fig. 5:**
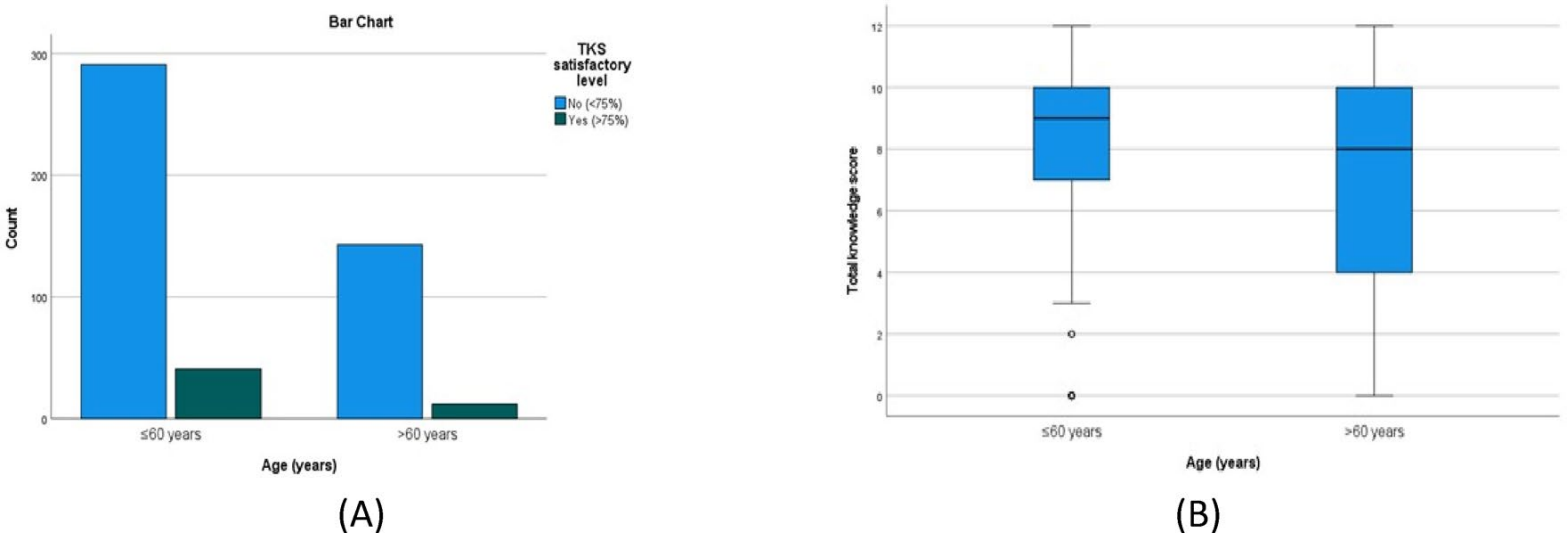
(**A**,**B**) A bar chart and a box plot that show the total knowledge score by age, equal to or less than 60, and more than 60. The green bar is satisfactory, and the blue bar is unsatisfactory.

There was a statistically significant higher TKS in females compared to males (*P* = 0.003), (Table 4; Fig. 3A and B), urban compared to rural residents (*P* = 0.002) (Table 5; Fig. 4A and B), the younger compared to the older age groups(*P* = < 0.001) (Table 6; Fig. 5A and B) and those with university/postgraduate level of education compared to illiterate/primary level education (p = < 0.001) (Table 7). Nonetheless, no statistically significant difference in the satisfactory level proportions across these groups (*P* = 0.475, 0.294, 0.128, 0.128, respectively).

**Table 4:** Total knowledge score and satisfactory knowledge level in male vs. female participants.

Characteristic	Male *N* = 159	Female *N* = 328	Test of significance	
Total knowledge score	Median (Q1-Q3)	Median (Q1-Q3)		**p-value**
	8 (4–10)	9 (7–10)		**0.003**
Satisfactory level	N (%)	N (%)	**χ** ^**2**^	**p-value**
< 75%	144 (90.6%)	290 (88.4%)	0.511	0.475
> 75%	15 (9.4%)	38 (11.6%)		

Mann-Whitney U-test for Total knowledge score and chi-square for satisfactory level according to gender.

**Table 5:** Total knowledge score and satisfactory knowledge level in rural vs. urban residence.

Characteristic	Rural	Urban	Test of significance	
Total knowledge score	Median (Q1-Q3)	Median (Q1-Q3)		**p-value**
	9 (5–10)	9 (8–10)		**0.002**
Satisfactory level	N (%)	N (%)	**χ** ^**2**^	**p-value**
< 75%	301(90.1%)	133(86.9%)	1.102	0.294
> 75%	33(9.9%)	20(13.1%)		

Mann-Whitney U-test for Total knowledge score and chi-square for satisfactory level according to residence area.

**Table 6:** Total knowledge score and satisfactory knowledge level in young (< 60 years) vs. old (> 60 years) participants.

Characteristic	< 60 years	> 60 years	Test of significance	
Total knowledge score	Median (Q1-Q3)	Median (Q1-Q3)		**p-value**
	9 (7–10)	8 (4–10)		**< 0.001**
Satisfactory level	N (%)	N (%)	**χ** ^**2**^	**p-value**
< 75%	291 (67.1%)	143 (32.9%)	2.313	0.128
> 75%	41 (77.4%)	12 (22.6%)		

Mann-Whitney U-test for Total knowledge score and chi-square for satisfactory level according to age.

**Table 7:** Total knowledge score and satisfactory knowledge level in different education levels.

Characteristic	Illiterate/primary	Middle/technical	University	Test of significance	
Total knowledge score	Median (Q1-Q3)	Median (Q1-Q3)	Median (Q1-Q3)		**p-value**
	8 (5–9) a	8 (9–10) b	10 (9–10) b		**< 0.001**
Satisfactory level	N (%)	N (%)	N (%)	**χ** ^**2**^	**p-value**
< 75%	234 (92.1%) a	150 (88.8%) a, b	50 (78.1%) b	10.367	**0.006**
> 75%	20 (7.9%) a	19 (11.2%) a, b	14 (21.9%) b		

Kruskal-Wallis H-test for Total knowledge score and chi-square for satisfactory level according to the level of education.

**Fig. 6:**
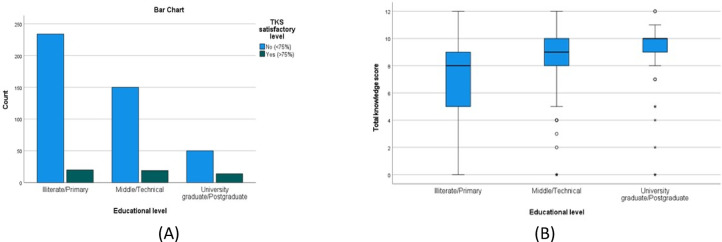
(**A**,**B**) A bar chart and a box plot that show the total knowledge score by education of illiterate/primary, middle/technical, and university graduate/postgraduate. The green bar is satisfactory, and the blue bar is unsatisfactory.

The binary logistic regression analysis was run to ascertain the effects of male sex, older age, rural residency, and lower education levels on the likelihood that participants will exhibit an unsatisfactory level of knowledge. Univariate analysis revealed that education level was a statistically significant predictor. It was entered in a multivariate analysis with age and residence. The model was statistically significant at χ^2^(4) of 10.268, and p-value of 0.036. Finally, participants with middle and illiterate/primary levels have 2.37- and 2.28-times higher odds of exhibiting an unsatisfactory level of knowledge compared with university graduate patients (AOR 2.37, 95% CI: 1.05–5.36, *P* = 0.038; and AOR 2.28, 95% CI: 1.36–7.91, *P* = 0.008, respectively) (Fig. 6; Table 8).

**Table 8:** Predictors of the likelihood of unsatisfactory knowledge level.

Risk factor	Univariate			Multivariate		
	COR	95% CI	*p*-value	AOR	95% CI	*p*-value
Sex						
Female	r(1)	r(1)	0.475	–	–	–
Male	1.26	0.67–2.36				
Age						
≤ 60 years	r(1)	r(1)	0.132	r(1)	r(1)	0.307
> 60 years	1.68	0.86–3.29		1.44	0.71–2.92	
Residence						
Urban	r(1)	r(1)	0.295	r(1)	r(1)	0.727
Rural	1.37	0.76–2.48		0.88	0.44–1.77	
Education level						
University	r(1)	r(1)		r(1)	r(1)	
Middle	2.21	1.03–4.73	**0.041**	2.37	1.05–5.36	**0.038**
Illiterate/primary	3.28	1.55–6.92	**0.002**	3.28	1.36–7.91	**0.008**

COR = crude odds ratio. AOR = adjusted odds ratio. CI = confidence interval. r(1) = reference category.

## Discussion

The focus in managing Stage IV solid malignancies is to alleviate symptoms and enhance quality of life. Addressing the treatment goals and early palliation in cancer patients has also been linked to better survival outcomes. Our study assessed 487 Stage IV cancer patients’ perception of treatment goals, revealing that 9.4% of the patients were unaware of their cancer diagnosis. We established a knowledge score and categorized only 53 patients as having a satisfactory level of knowledge and understanding about their diseases and treatment intent. Recognizing factors linked to misconceptions about treatment goals can help in formulating clinical strategies. Our study identified that education level was the sole statistically significant independent predictor of unsatisfactory level of knowledge, while factors like age, gender, and residential area showed significance in univariate analysis, they did not maintain independence in the multivariate analysis.

This misperception of treatment objectives is reported in numerous studies across different geographical locations. A study conducted in Harvard Cancer Center revealed that 38% of the patients had misunderstandings and discrepancies in the perception of their treatment goals. Interestingly, it also showed that there were no significant associations between patient education, race, ethnicity, relationship status, or time since diagnosis of incurable cancer and reported QoL^22^.

Another study indicated that only half of the patients acknowledged their terminal illness status, with 75% believing that their treatment aimed to cure and prolong life expectancy^23^. A study by Alsirafy et al. reported significant overestimation of treatment goals, especially in patients treated with immunotherapy. On the other hand, it found no correlation between age, race, or education level and the perception of therapeutic benefit^19^. The discrepancy in these findings in our study and the others could be explained by variations in literacy rates and the patient-physician relationship, which is influenced by cultural factors and population characteristics.

The association between education level and knowledge may be influenced by unmeasured factors such as the quality of patient counselling, community culture, family factors, and access to information. These variables may act as confounders and contribute to variations in patient understanding beyond classic education level. While education level, residency, and old age were reported in our cohort to be significant factors affecting the knowledge, the multivariate analysis identifies education level as the main independent factor. This may be simply because lower education levels are more common in rural areas and among older age groups.

While patients’ comprehension of their disease’s nature and treatment intent is a basic and essential element of the informed consent process, some cultures, families, and even health care providers prefer to intentionally withhold prognostic information. Some argue that the truth will lead to increased anxiety, patient depression, and worse QOL. Prior studies have shown that patients who acknowledge the terminal nature of their illness report worse QoL and psychological distress^23^. However, other studies found that when compared to patients who remained unaware of their disease status, those who were informed demonstrated significantly better role, emotional, and social functioning, as well as fewer symptoms of fatigue, loss of appetite, constipation, financial difficulties, and anxiety^24,25^. Implementing coping strategies can improve patients’ psychological wellbeing^22,23^.

The misunderstanding of treatment intent may arise when some patients express their hopes or wishes rather than accurately understanding their illness. It is important to distinguish between the true lack of knowledge and the wrong answers representing false hope, denial, or psychological defense. Some responses classified as improper knowledge of the treatment palliative intent may, in fact, reflect one of these possibilities. Also, this misperception is not exclusive to solid tumor patients only. A study done among older adult AML patients showed that 90% of patients stated that they were “somewhat” or “very likely” to be cured of their AML, but only 31% of those patients were given an estimate by oncologists that they would be cured. The patients also overestimated their treatment-related mortality^26^. Additionally, the misunderstanding is not limited to the older patient population; a study assessed the perception of treatment goals among the pediatric patient population, and their caregivers found that they tend to have a falsely optimistic view. Most patients (78%) and parents (85%) reported belief in a remarkably high chance of cure. It is worth noting that prognostic optimism varied significantly by type of cancer; patients with solid tumors had higher levels of optimism, and lymphoma patients were most frequently accurate^27^.

Our study has some limitations. Firstly, and more importantly, is the lack of questionnaire validation. This questionnaire was customized to address this specific research gap, with no other validated questionnaires available. It was designed to reflect both clinical and objective variables and to reduce selection and data bias. To overcome this limitation, the questionnaire was reviewed by oncology specialists before the research began. But these findings must be interpreted with caution, given the exploratory nature of the questionnaire. Secondly, dichotomization of the Total Knowledge Score may have resulted in information loss and affected the statistical power. Thirdly, excluding patients who denied having cancer may have led to an underestimation of the true level of misunderstanding. Fourthly, the data were based on self-reported responses, making them subject to recall and social desirability bias, particularly in a cultural context where discussing cancer openly may be sensitive. Fifthly, using both self-reported and physician-administered questionnaires introduces potential interviewer and social desirability bias, particularly in illiterate patients. This means the strong association described between lower education levels and lower knowledge scores should be interpreted while keeping in mind this potential confounding bias. Sixthly, it is crucial to acknowledge that palliative care may occasionally help prolong survival. Therefore, the patient’s belief that a treatment could prolong their life may lead to an optimistic view of the treatment’s goals. This could have caused our knowledge assessment to be partially altered. Seventhly, the study did not assess psychological factors such as anxiety, depression, or coping mechanisms, nor did it evaluate physician-related communication variables, both of which may significantly influence patients’ perceptions.

Future interventional studies with structured educational interventions can be used to assess the improvement of patients’ understanding. A follow-up assessment to evaluate patients’ outcomes is needed to correlate it with the patient’s knowledge level. Furthermore, facilitating strong and effective communication between physicians and patients is fundamental for conveying information and ensuring that patients have a full comprehension of their disease status.

## Conclusion

Our findings suggest a substantial gap in understanding of disease nature and treatment goals among Egyptian cancer patients, particularly those with lower education, rural residency, and advanced age. Improved patient education and communication protocols designed to cope with the local culture are essential to ensure shared decision-making and improve quality of life. These findings must be interpreted with caution, given the exploratory nature of the questionnaire. Further research using validated psychometric tools is mandatory to confirm these findings.

## Data Availability

The datasets generated and analysed during the current study are available from the corresponding author on reasonable request.
